# Peak Running Velocity or Critical Speed Under Field Conditions: Which Best Predicts 5-km Running Performance in Recreational Runners?

**DOI:** 10.3389/fphys.2021.680790

**Published:** 2021-07-06

**Authors:** Diogo Hilgemberg Figueiredo, Diego Hilgemberg Figueiredo, Francisco de Assis Manoel, Fabiana Andrade Machado

**Affiliations:** ^1^Associate Post-graduate Program in Physical Education, Department of Physical Education, State University of Maringá, Maringá, Brazil; ^2^Department of Physical Education, Cesumar University, Maringá, Brazil; ^3^Department of Physical Education, State University of Maringá, Maringá, Brazil; ^4^Post-graduate Program of Physiological Sciences, Department of Physiological Sciences, State University of Maringá, Maringá, Brazil

**Keywords:** prediction, performance, running, endurance, exercise test

## Abstract

This study aimed to examine which variable, between the peak running velocity determined on the track field (*V*_peak_TF_) and critical speed (CS), is the best predictor of the 5-km running performance in recreational runners. Twenty-five males performed three tests to determine the *V*_peak_TF_, CS, and 5-km running performance on the track field, with a minimal interval of 48 h between each test. The *V*_peak___*TF*_ protocol started with a velocity of 8 km⋅h^–1^, followed by an increase of 1 km⋅h^–1^ every 3 min until volitional exhaustion, which was controlled by sound signals, with cones at every 25 m indicating when the participants were required to pass the cone’s position to maintain the required velocity. The participants performed three time trials (TTs) (1: 2,600 m; 2: 1,800 m; and 3: 1,000 m) on the same day, with a 30-min rest period to determine the CS through the combinations of three (CS_1_,_2_,_3_) and two TTs (CS_1_,_2_, CS_1_,_3_, and CS_2_,_3_). The 5-km running performance time was recorded to determine the test duration, and the mean velocity (MV) was calculated. There was a significant difference observed between the *V*_peak_TF_ and the MV 5-km running performance. However, no differences were found between the CS values and the MV 5-km running performance. A correlation was observed between the *V*_peak_TF_ (*R* = −0.90), CS_1_,_2_,_3_ (*R* = −0.95), CS_1_,_3_ (*R* = −0.95), and the 5-km running performance time. Linear regression indicated that the *V*_peak_TF_ (*R*^2^ = 0.82), CS_1_,_2_,_3_ (*R*^2^ = 0.90), and CS_1_,_3_ (*R*^2^ = 0.90) significantly predicted the 5-km running performance time. The CS results showed a higher predictive power for the 5-km running performance, slightly better than the *V*_peak_TF_. Also, CS_1_,_2_,_3_ and the CS_1_,_3_ presented the highest predictive power for the 5-km running performance of recreational runners.

## Introduction

Millions of recreational runners participate in long-distance running competitions (i.e., 5 and 10 km) each year, being consistently considered among the most popular distances and with the greatest number of competitions, even greater than marathons (Cushman et al., 2014; Vickers and Vertosick, 2016). Therefore, it is important to apply test protocols that assess the aerobic capacity to accurately predict the running performance, to which aerobic metabolism contributes about 95% of the total energy expenditure (Busso and Chatagnon, 2006). It is possible through these test protocols to be able to identify the physiological and performance variables that might be used to improve the prediction of the runners’ performances, such as the maximal oxygen uptake (${\dot{\text{V}}\text{O}}_{\text{2max}}$), the velocity of ${\dot{\text{V}}\text{O}}_{\text{2max}}$ occurrence (v${\dot{\text{V}}\text{O}}_{\text{2max}}$), running economy (RE), the responses associated with the blood lactate concentrations during exercise [i.e., lactate threshold (LT), anaerobic threshold (AnT), and maximal lactate steady state (MLSS)], peak running velocity (*V*_peak_), and critical speed (CS) (Machado et al., 2013; da Silva et al., 2015; Nimmerichter et al., 2016).

Among these variables, the *V*_peak_ and CS stand out, which can be determined in simple, objective, and sensitive protocols that do not require the use and handling of expensive and delicate equipment or invasive techniques, considered accessible and of great practical application (Jones and Poole, 2009).

*V*_peak_ is defined as the highest effort intensity achieved during an incremental test until the maximum volitional exhaustion (Noakes et al., 1990), which is considered a strong predictor of endurance running performance and presents high correlation with the 3–90 km events (Slattery et al., 2006; Stratton et al., 2009; Machado et al., 2013). For instance, Machado et al. (2013) reported high correlation (*R* = 0.95) and predictive power (*R*^2^ = 0.91) between the *V*_peak_ determined on the incremental treadmill test protocol (*V*_peak___T_) with a 3-min stage duration, defined according to Kuipers et al. (2003), and the 5-km running performance of recreational runners.

CS represents the intensity of effort (e.g., running speed) that can be maintained for an extended period (≈30–60 min) without a continual rise in systemic [e.g., blood lactate concentration (La) and oxygen uptake (${\dot{\text{V}}\text{O}}_{\text{2max}}$)] and intramuscular metabolism (e.g., pH and phosphocreatine concentration) homeostasis (Jones et al., 2008, 2010; Poole et al., 2016; Jones and Vanhatalo, 2017). This concept is based on the hyperbolic relation between the predetermined intensities of effort (i.e., distance or running speed) and the time it takes to reach exhaustion (i.e., time limit- *t*_*lim*_) (Hughson et al., 1984; Hill, 1993). Previous studies have also investigated the use of CS for running performance prediction in distances ranging from 40 m to longer distances such as that of a marathon (Kranenburg and Smith, 1996; Florence and Weir, 1997; Nimmerichter et al., 2016). A recent study involving trained endurance athletes has observed higher correlations (*R* = −0.79 and 0.82) and predictive power (*R*^2^ = 0.64 and 0.67) between the CS estimated on the treadmill test protocol performed on the same day with time and the mean velocity (MV) 5-km running performance, respectively, suggesting that CS is valuable for predicting performance compared to ${\dot{\text{V}}\text{O}}_{\text{2max}}$ (Nimmerichter et al., 2016).

Nevertheless, to predict the endurance of running performances, the determination of *V*_peak___T_ and the estimation of CS were exclusively performed under laboratory conditions (Stratton et al., 2009; Machado et al., 2013; Nimmerichter et al., 2016), which do not provide ecological validity due to the different characteristics of a treadmill and track field running regarding propulsion, overcoming air resistance, inertia, and gait pattern that might affect the utilization of treadmill-derived measures into field conditions (Van Caekenberghe et al., 2013). Tests performed on a track field are more applicable due to the higher specificity to the sports’ performance, which can be easily integrated into a daily training routine and, therefore, are less time-consuming than laboratory tests. Furthermore, the development of knowledge concerning the prediction of the 5-km running performance that underlies these variables present on the track field will enable greater specificity on the prescription of training intensities and could also provide practitioners and their coaches the optimal pacing and tactical strategies that will allow improvements on their competitive results.

At the moment, there is no consensus on the best predictor variable (*V*_peak_ or CS) contributor determined on the track field relative to the 5-km running performance. Thus, this study aimed to examine which variable, between the peak running velocity determined on the track field (*V*_peak_TF_) and the critical speed (CS), is the best predictor of the 5-km running performance in recreational runners. The study’s hypothesis is that *V*_peak_TF_ has a higher predictive power for the 5-km running performance than does the CS, given that *V*_peak_ is the “determined” velocity associated with the ${\dot{\text{V}}\text{O}}_{\text{2max}}$ established through an incremental protocol, while CS is “estimated” through linear regression using mathematical models with a constant distance path protocol.

## Materials and Methods

### Participants

Twenty-five male recreational runners, regional and local level competitors (mean ± SD: age = 28.6 ± 4.7 years, height = 176.2 ± 9.7 cm, body mass = 78.5 ± 10.4 kg, relative lean mass = 89.3 ± 4.5%, relative adipose mass = 10.7 ± 4.5%), with a 5-km running performance time of 25.3 ± 3.0 min and MV of 12.0 ± 1.3 km⋅h^–1^ (which represented 49.8% of the MV from the world record) were recruited as the participants in this study.

All participants were physically active with a training running experience of at least 2 years and had a training frequency of 3.0 ± 0.7 days⋅week^–1^, with an average distance of 24.4 ± 7.3 km⋅week^–1^. They presented medical clearance to perform exhaustive physical tests and reported no use of nutritional ergogenic supplements for the duration of the study. To include the participant’s data in the final analysis, the following requirement was adopted: Presenting a 5-km running performance time between 21.4 and 32.6 min (Machado et al., 2013; Vickers and Vertosick, 2016; Peserico et al., 2019). The participants were informed that they were free to withdraw from the study at any time. Prior to testing, a written consent form was obtained from all participants. The experimental protocol was approved by the local Human Research Ethics Committee (no. 2.698.091/2018).

### Design

After the familiarization process with the track field test protocols, each participant performed three randomly ordered tests to determine the *V*_peak_TF_, the CS, and the 5-km running performance on the official outdoor track field (400 m) at the same time of the day under similar climatic conditions (temperature = 25–29 °C and relative humidity = 60–75%), with an interval of 48 h between each test. They were instructed to report for testing well rested, well hydrated, and wearing lightweight comfortable clothing and also to avoid eating 2 h before the maximal exercise tests, to abstain from caffeine and alcohol, and to refrain from strenuous exercise for 24 h before testing (Machado et al., 2013). All of the participants were verbally encouraged throughout the tests, and mineral water was provided *ad libitum* so that the participants could hydrate themselves, as they were used to do in long-distance races.

### Determination of *V*_peak_ on the Track Field

The protocol used to determine *V*_peak_TF_ was the same one used for the determination of *V*_peak_T_ (Machado et al., 2013). After a warm-up, consisting of walking at 6 km⋅h^–1^ for 3 min, the protocol started with an initial velocity of 8 km⋅h^–1^, followed by an increase of 1 km⋅h^–1^ every 3 min (Machado et al., 2013). The velocity during the protocol on the track field was controlled by sound signals, with cones at every 25 m, indicating when the participants were required to pass the cone’s position to maintain the required velocity (Léger and Boucher, 1980). The protocol ended when the participants reached volitional exhaustion (i.e., the participant was unable to continue running) or when the evaluator identified that the participants failed to cross the cone line with one of two feet on three consecutive occasions (Léger and Boucher, 1980). If the last stage was not completed, *V*_peak_TF_ was calculated with the partial time remaining in the last stage according to the equation: *V*_peak_TF_ = *V*_*complete*_ + (Inc × *t*/*T*), where *V*_*complete*_ is the running velocity of the last complete stage, Inc is the velocity increment (i.e., 1 km⋅h^–1^), *t* is the number of seconds sustained during the incomplete stage, and *T* is the number of seconds required to complete a stage (i.e., 180 s) (Kuipers et al., 2003).

### Determination of Critical Speed

Each participant performed three time trials (TTs) on the track field (1: 2,600 m; 2: 1,800 m; and 3: 1,000 m). These TTs were selected according to Galbraith et al. (2011) and Hughson et al. (1984) to result in completion times between 3 and 12 min before volitional exhaustion. Consistent with Triska et al. (2017) and Galbraith et al. (2011), the sequence of TTs was conducted in the order of the longest to the shortest effort, on the same day, with a 30-min rest period to ensure a fully reconstituted *D*′ (maximum distance covered above the CS). The participants completed a 5-min self-paced low-intensity warm-up exercise and were encouraged to cover the set TTs as quickly as possible; time was measured using a stopwatch (Galbraith et al., 2014). The CS was estimated through a linear regression between the distance run (*d*) and *t*_*lim*_ using the *d* = (CS × *t*_*lim*_) + *D*′ model, where *d* is the distance run (in meters), CS the critical speed (in meters per second), *t*_*lim*_ the time to exhaustion (in seconds), and *D*′ is the maximum distance covered (in meters) above the CS (Hughson et al., 1984; Galbraith et al., 2011). CS was estimated through the combinations of three (CS_1_,_2_,_3_) and two TTs (CS_1_,_2_, CS_1_,_3_, and CS_2_,_3_).

### 5-km Running Performance

The 5-km running performance was preceded by a self-selected warm-up of 10 min. The participants freely choose their pacing strategy during this performance and were encouraged to cover the set distance as quickly as possible on the track field. The 5-km running performance time for each participant was recorded and registered by the evaluator using a stopwatch to determine the test duration, and MV was calculated by dividing the total distance by the trial duration. No information on the elapsed time was provided for the participants.

### Statistical Analysis

The Statistical Package for the Social Sciences (SPSS^®^ v25.0 for Windows, Inc., Chicago, IL, United States) was used to conduct the analysis. The normality assumption was verified using the Shapiro–Wilk test, and the results are presented as the mean ± SD. Sphericity was tested using Mauchly’s test. Greenhouse–Geisser corrections were made when the assumptions of sphericity were violated. One-way ANOVA for repeated measures followed by Bonferroni *post hoc* test was used to evaluate the differences between *V*_peak_TF_ and CS that resulted from the different time trial (TT) combinations and the 5-km running performance. Separate linear regression models were fit to establish Pearson’s product-moment correlations (*R*), coefficients of determination (*R*^2^), and the standard error of the estimate (SEE) to examine the goodness of fit of the univariate relation between the *V*_peak_TF_ and CS that resulted from the different TT combinations (independent variables) and the 5-km running performance (dependent variable). The correlation coefficients (*R*) were interpreted using the following qualitative descriptors: Trivial (<0.1), small (<0.3), moderate (0.3–0.5), large (0.5–0.7), very large (0.7–0.9), nearly perfect (>0.9), and perfect (1.0) (Hopkins et al., 2009). Absolute agreement and the overall mean bias between CS_1_,_2_,_3_ with CS_1_,_2_, CS_1_,_3_, and CS_2_,_3_ were determined using limits of agreement (LoA) analysis (Bland and Altman, 1986). Furthermore, SEE was also calculated to show any error between the CS results from the different TT combinations. The significance level was set at *P* < 0.05 for all statistical analyses.

## Results

The *V*_peak_TF_, the CS values estimated from the different TT combinations, and the MV for the 5-km running performance are shown in Table 1. There were significant differences between the *V*_peak_TF_ and CS values and the MV for the 5-km running performance. However, there were no differences between the CS values as well as between the CS values with MV for the 5-km running performance.

**TABLE 1:** Mean ± SD and range obtained from the *V*_peak_TF_, CS values estimated through different TT combinations, and the MV for the 5-km running performance (*n* = 25).

Variable	Mean ± SD (km⋅h^–1^)	Range (km⋅h^–1^)
*V*_peak_TF_	13.7 ± 1.1	11.0–15.9
CS_1_,_2_,_3_	12.1 ± 1.4*	8.6–13.8
CS_1_,_2_	12.5 ± 1.7*	8.0–15.4
CS_1_,_3_	12.1 ± 1.4*	8.7–13.8
CS_2_,_3_	11.7 ± 1.4*	7.4–13.7
MV 5-km running performance	12.0 ± 1.3*	9.2–14.1

V_*peak_TF*_, Peak running velocity determined on the track field; CS_1;2;3,_ Critical speed at 2,600, 1,800 and 1,000 m; CS_1;2,_ Critical speed at 2,600 and 1,800 m; CS_1;3,_ Critical speed at 2,600 and 1,000 m; CS_2;3,_ Critical speed at 1,800 and 1,000 m; MV, Mean velocity. *P < 0.001 in relation to *V*_*peak*___*TF*_.

**FIGURE 1:**
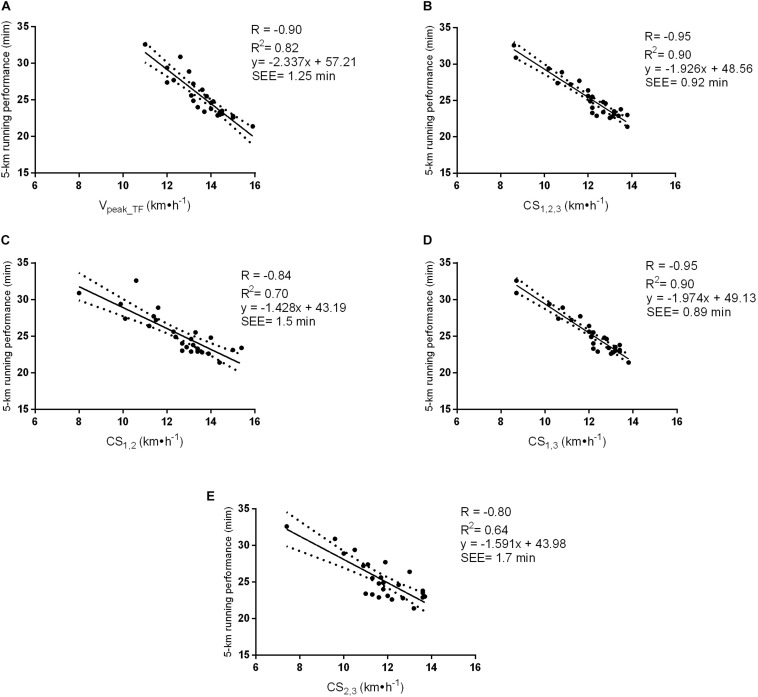
Correlation and linear regression between the (V_peak_TF_) **(A)**, CS_1_,_2_,_3_ **(B)**, CS_1_,_2_ **(C)**, CS_1_,_3_ **(D)**, and CS_2_,_3_ **(E)** with the 5-km running performance time in minutes (*n* = 25). SEE: standard error of the estimate; V_peak_TF_: Peak running velocity determined on the track field; CS_1,2,3_: Critical speed at 2,600, 1,800 and 1,000 m; CS_1,2_: Critical speed at 2,600 and 1,800 m; CS_1,3_: Critical speed at 2,600 and 1,000 m; CS_2,3_: Critical speed at 1,800 and 1,000 m.

Figure 1 shows the relation between each independent variable (the *V*_peak_TF_ and CS values estimated from the different TT combinations) and the 5-km running performance time. The *V*_peak_TF_ (*R* = −0.90) and the CS (*R* = −0.80 to −0.95) values showed high and negative correlations with the 5-km running performance time. Linear regression analysis indicated that the *V*_peak_TF_, CS_1_,_2_,_3_, CS_1_,_2_, CS_1_,_3_, and CS_2_,_3_ significantly predicted 82, 90, 70, 90, and 64% of the variance in the 5-km running performance time, respectively.

**FIGURE 2:**
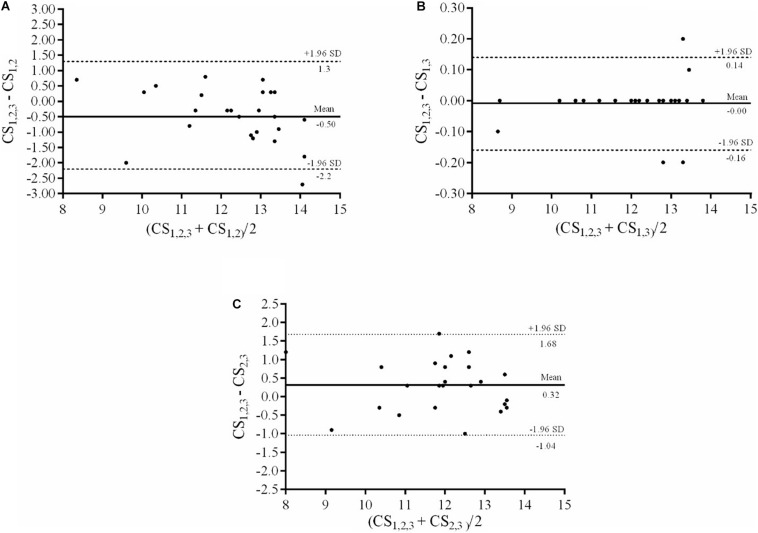
Bland–Altman plots of the differences between CS_1_,_2_,_3_ and CS_1_,_2_ **(A)**, CS_1_,_3_ **(B)**, and CS_2_,_3_ **(C)**. *Solid horizontal lines* represent the mean bias, while *dashed lines* represent the lower and upper 95% limits of agreement. CS_1,2,3_: Critical speed at 2,600, 1,800 and 1,000 m; CS_1,2_: Critical speed at 2,600 and 1,800 m; CS_1,3_: Critical speed at 2,600 and 1,000 m; CS_2,3_: Critical speed at 1,800 and 1,000 m.

The Bland–Altman plots of the differences between CS_1_,_2_,_3_ and CS_1_,_2_, CS_1_,_3_, and CS_2_,_3_ are presented in Figure 2. The results revealed the highest agreement (i.e., the overall mean bias was least and the 95% LoA narrowest) in the comparison between CS_1_,_2_,_3_ and CS_1_,_3_. In comparison to CS_1_,_2_,_3_, CS_1_,_3_ showed a SEE of 0.08 km⋅h^–1^ and CS_1_,_2_ and the CS_2_,_3_ showed SEEs of 0.76 and 0.67 km⋅h^–1^, respectively.

## Discussion

The present study aimed to examine which variable, between the peak running velocity determined on the track field (*V*_peak_TF_) and the critical speed (CS), is the best predictor of the 5-km running performance in recreational runners. The main finding was that CS showed a higher correlation and predictive power for the 5-km running performance, slightly better than that of *V*_peak_TF_, which is contrary to the initial hypothesis. Also, the CS values estimated through three TTs (i.e., CS_1_,_2_,_3_) and the combination of the shortest and the longest TTs (i.e., CS_1_,_3_) presented the highest correlation and predictive power for this performance in recreational runners.

This is the first study that demonstrated the relation between both the *V*_peak_TF_ and CS determined on the track field and the 5-km running performance in recreational runners. These results are in accordance with previous studies that demonstrated a higher correlation and predictive power between the *V*_peak___T_ and CS determined from a treadmill test protocol performed on the same day and the 5-km running performance in untrained volunteers (Stratton et al., 2009), recreational runners (Machado et al., 2013), and endurance athletes (Nimmerichter et al., 2016). However, it must be emphasized that, unlike the method used in this study, Stratton et al. (2009) determined the *V*_peak___T_ using a different protocol, not only the duration of the tests but also the initial speed, and the aforementioned studies also conducted both the CS and *V*_peak_ protocols under laboratory conditions, which could explain the high predictive power for both variables determined on the track field in our study, reflecting the demands of competitive reality and endurance training, as well as the highest ecological validity.

Although the *V*_peak_TF_ and CS determined on the track field have been shown to be effective predictors of the 5-km running performance, CS_1_,_2_,_3_ and CS_1_,_3_ demonstrated higher correlations and predictive power that were slightly better than those of *V*_peak_TF_. This result may be explained by the fact that all values of CS were similar to the MV for the 5-km running performance in the present study (Table 1), thus suggesting that the participants may have performed the 5-km running performance at intensities close to 100% of the CS. Previous studies have reported that the *t*_*lim*_ at an intensity associated with the CS during running could be sustained for less than 30 min (Pepper et al., 1992; Bull et al., 2008). Interestingly, this is very similar to the average time performed during the 5-km running performance in this study (25.3 ± 3.0 min), confirming that the CS may be held for the length of time taken to complete this performance in recreational runners. However, unlike the CS, previous studies have shown that the *t*_*lim*_ at an intensity associated with 100% of the *V*_peak_ during a treadmill protocol could be sustained for less than 7 min by recreational runners (da Silva et al., 2015; Peserico et al., 2019). Thus, we suggest that the similarity between the CS and the MV for the 5-km running performance may in part explain the higher power of the CS to predict performance.

The present study also showed that the CS estimated through three TTs (i.e., CS_1_,_2_,_3_) was not different from the combination of the shortest and the longest TTs (i.e., CS_1_,_3_) (Table 1). These data are in agreement with those of Smith et al. (2011) showing that the CS estimated from the shortest and the longest *t*_*lim*_ trials (i.e., 110 and 90% v${\dot{\text{V}}\text{O}}_{\text{2max}}$, respectively) can produce similar estimates and the lowest standard error of the mean (SEM) when compared with the CS data from the four *t*_*lim*_ trials (i.e., 90, 100, 105, and 110% v${\dot{\text{V}}\text{O}}_{\text{2max}}$) on a treadmill in moderately trained runners, respectively. Similarly, Kordi et al. (2019), using a similar method to that proposed in our study, reported that the CS values estimated from the shortest and the longest TTs (i.e., 3,600 and 1,200 m, respectively) were similar and also showed an overall lowest mean bias and SEE in comparison with the CS estimated through three TTs (i.e., 3,600, 2,400, and 1,200 m) in experienced, highly trained runners.

Nonetheless, Maturana et al. (2018) demonstrated that an accurate estimation of critical power (CP) in cyclists was achieved when TTs with longer durations were included when compared with five predictive TTs. However, when only short TTs (i.e., less than 10 min) are used, it might result in a higher or a lower estimation of the CP, leading to poor agreement with the CP estimated from a predetermined criterion method (i.e., five TTs) (Maturana et al., 2018). Thus, longer TTs should be included in order to model the CP that more realistically predicts the upper boundary of sustainable endurance exercise, which required a high level of ${\dot{\text{V}}\text{O}}_{\text{2max}}$ (Morton, 2006). In addition, Bishop et al. (1998) showed that the estimation of CP was higher when the TT durations became shorter, and the opposite was true when the TT durations were lengthened.

Thus, it is necessary that the duration and the number of these distances are carefully selected. Previous studies have recommended the TTs range between 2 and 15 min (Hill, 1993; Bishop et al., 1998), with a minimum of 5 min difference between the shortest and the longest TTs (Housh et al., 1990) to help participants with slower ${\dot{\text{V}}\text{O}}_{\text{2}}$ kinetics and also to ensure attainment of the ${\dot{\text{V}}\text{O}}_{\text{2max}}$ and discharging *D*′ at the end of each exhaustive TT (Simpson and Kordi, 2017). In addition, previous studies have shown that the total number of TTs required to estimate the CS ranges between three and five (Kranenburg and Smith, 1996; Galbraith et al., 2014; Triska et al., 2018), although it is usual for at least three TTs to be performed, especially in non-athletes (Karsten et al., 2014), which is in agreement with the number and the duration range used in the present study.

Although our results suggest that CS_1_,_2_,_3_ and the CS_1_,_3_ accounted for the majority of the total variance associated with predicting the 5-km running performance time when compared to the *V*_peak_TF_, this should be carefully interpreted due to the slight differences in the predictive power found between these variables and the 5-km running performance time (Figure 1), with the *V*_peak_TF_ being almost as good as the CS_1_,_2_,_3_ and CS_1_,_3_. Nevertheless, previous studies have shown that *V*_peak___T_ is highly reliable (Peserico et al., 2014) and has been reported to be a valid measure to prescribe and evaluate improvements in the endurance performance of recreational runners (Peserico et al., 2019).

In contrast, the lack of reliability on the test for CS that resulted from the different TT combinations in recreational runners can be considered a potential limitation. This may arguably increase the potential use of *V*_peak_TF_ compared to CS in predicting the 5-km running performance of recreational runners. Thus, further studies are required to examine whether the reliability of the CS that resulted from the different TT combinations has as high reliability as the *V*_peak___T_ in recreational runners in order to effectively predict endurance performance as well as for the prescription and analysis of the training effects.

Therefore, we conclude that the CS results showed a higher predictive power for the 5-km running performance, slightly better than that of *V*_peak_TF_. Also, CS_1_,_2_,_3_ and the CS_1_,_3_ presented the highest predictive power for the 5-km running performance of recreational runners.

## Practical Application

Understanding the relation between the *V*_peak_TF_ and CS determined on the track field with the 5-km running performance would enable coaches, practitioners, and endurance runners to increase specificity in the training methods, which will allow improvements of their competitive results. These variables could be considered a more practical way to evaluate and monitor the effects of endurance training, as well as in elucidating a more homogeneous response when used to prescribe adequate exercise intensities compared to other physiological parameters such as the ${\dot{\text{V}}\text{O}}_{\text{2max}}$, v${\dot{\text{V}}\text{O}}_{\text{2max}}$, and LT. We suggest that a well-structured and periodized training program should take these variables into consideration on the track field as a way of improving the 5-km running performance of recreational runners.

Moreover, the proposed equations can be used to predict the 5-km running performance from the results of the *V*_peak_TF_, the CS_1_,_2_,_3_, and the CS_1_,_3_ estimated on the track field, which has an easy accessibility for coaches, practitioners, and endurance runners. Finally, the estimation of the CS using the combination of the shortest and the longest TTs (i.e., CS_1_,_3_) is extremely relevant regarding time efficiency and employing applicability setting (i.e., one training session), which can minimize the time commitment of practitioners and endurance runners during assessments.

## Data Availability Statement

The raw data supporting the conclusions of this article will be made available by the authors, without undue reservation.

## Ethics Statement

The studies involving human participants were reviewed and approved by the Human Research Ethics Committee, State of University of Maringá. The patients/participants provided their written informed consent to participate in this study.

## Author Contributions

DioF, DieF, FdAM, and FAM performed material preparation, data collection, and analysis. DioF wrote the first draft of the manuscript. All authors commented on previous versions of the manuscript, read and approved the final version of the manuscript, and contributed to the study conception and design.

## Conflict of Interest

The authors declare that the research was conducted in the absence of any commercial or financial relationships that could be construed as a potential conflict of interest.
